# EXAMINING THE ASSOCIATION BETWEEN MULTIMORBIDITY PATTERNS AND MORTALITY RISK AMONG PEOPLE WITH DEMENTIA

**DOI:** 10.1093/geroni/igae098.0448

**Published:** 2024-12-31

**Authors:** Hao Luo, Jiayi Zhou

**Affiliations:** The University of Hong Kong, Hong Kong, China (People’s Republic); The University of Hong Kong, Hong Kong, China (People’s Republic)

## Abstract

People living with dementia often live with other diseases. Understanding how other diseases co-occur (‘multimorbidity’, concurrence of two or more diseases) in a person with dementia is important as it can inform a tailored approach to care. This study aimed to identify multimorbidity patterns among patients with and without dementia and examine how these patterns were associated with mortality. We identified dementia patients diagnosed at 65+ between 2007 and 2017 from electronic medical records from all public hospitals in Hong Kong. Each patient was 1:1 matched with patients without dementia by age, sex and index date and followed until death or 31 Dec 2018, whichever was earlier. A total of 156,710 individuals (mean age [SD]: 83.26 [7.28]; 59.6% females) were included. Seven multimorbidity patterns were identified from latent class analysis in the total sample, and similar patterns were identified separately for patients with and without dementia. The pattern characterized by “dyslipidemia and neurological diseases” was most likely to coexist with dementia. Among patients with dementia, compared to the “unspecific” pattern (characterized by the lack of defining diseases), all other patterns were associated with an increased risk of mortality. The highest risk was observed in the “clinically complex” pattern [HR 1.65 (95% CI: 1.60, 1.70)], followed by the “respiratory disease” [HR 1.51 (95% CI: 1.45, 1.57)] and “heart disease” patterns [HR 1.40 (95% CI: 1.36, 1.45)]. The results highlight the importance of taking specific combinations of chronic conditions into account when tailoring the clinical and care approach to people with dementia.

